# Community Strengths and Challenges Related to Opioid Use Disorder in Rural Counties of East Tennessee

**DOI:** 10.13023/jah.0401.04

**Published:** 2022-02-13

**Authors:** Ashlyn N. Schwartz, Zeruiah V. Buchanan, Laurie L. Meschke

**Affiliations:** Department of Public Health, The University of Tennessee, Knoxville; Arizona Health Care Cost Containment System; Department of Public Health, The University of Tennessee, Knoxville

**Keywords:** Appalachia, opioid use disorder, stigma, community-based participatory research, rural health

## Abstract

**Introduction:**

Appalachia, particularly Rural East Tennessee, has been and continues to be disproportionately impacted by opioid use disorder and its many tragic ramifications.

**Purpose:**

Community-engaged strategies can inform and support the development of relevant prevention efforts. Hence, people connected to a ten-county rural Appalachian region in East Tennessee were asked to identify and prioritize strengths and challenges related to opioid use disorder (OUD).

**Methods:**

Adult community members (n=577) completed a brief survey administered across 11 days in 2019.

**Results:**

Of the respondents, 85.3% never had been addicted to opioids, but 74.0% had someone close to them with OUD. The most frequently selected community strength was support for people with OUD to seek help and the most frequently selected challenge was lack of treatment and recovery services. People with personal OUD experience reported significantly higher mean levels of OUD-related stigma compared to persons without such experience.

**Implications:**

The number of respondents indicates a local concern and commitment related to OUD in rural Appalachia. The findings prioritized areas of focus—both in needs to be addressed and strengths on which to capitalize. These community insights will guide the selection and development of OUD-related overdose prevention for this region.

## INTRODUCTION

The East Tennessee Appalachian region is disproportionately burdened by opioid use disorder (OUD), especially within rural communities.^1^ To effectively identify and prioritize factors related to long-standing challenges of OUD in this region, community-based research strategies are critical. These data inform the design and implementation of culturally responsive prevention efforts.^2^

The Rural Communities Opioid Response Program for East Tennessee Consortium (RCORP-ETC) is co-led by the University of Tennessee, Knoxville, and community members from ten rural Tennessee counties. Within the RCORP-ETC region, a collision of high proportions of physical laborers, insufficient regulatory oversight, and targeted marketing by pharmaceutical companies has led to high availability of prescription pain medications.^3^ Moreover, the systemic challenges of restricted economic opportunities, unemployment, inadequate transportation, remote medical services, and limited treatment services in the RCORP-ETC region have intensified the prevalence and severity of OUD in this region.^4^ While OUD-related overdose continues to increase,^5^ treatment remains low due to insufficient resources, inadequate access to care, and high levels of stigma.^6^

Stigma, in particular, is a pervasive social challenge that hinders OUD prevention and treatment with its promotion of social disapproval and discrimination.^7^ Perceived stigma from community members can negatively influence persons with OUD, resulting in psychological distress, poorer quality of life, continued substance use, and reduced engagement with OUD treatment.^8^ Stigma’s effect on OUD treatment may be more pronounced in small, rural communities. For example, rural clinicians have indicated little willingness to provide treatment services due to community OUD stigma,^9^ and community members have reported concerns about being observed while attending treatment centers.^10^

Despite the importance of community support and compassion in the eradication of OUD, community members’ perspectives and experiences associated with OUD are poorly understood. To address this gap, we developed an evidence-based assessment tool to understand the priority concerns and strengths of community members, and how these may differ by perceived stigma and OUD experience.

## METHODS

All persons aged 18 or older who worked, lived, or played in the ten-county region (i.e., Campbell, Claiborne, Cocke, Grainger, Hamblen, Jefferson, Morgan, Roane, Scott, and Union) of East Tennessee were eligible to answer the paper or online survey. Given survey development, community vetting, and funder reporting timelines, 11 days were allotted for survey completion in 2019. Collecting a convenience sample, the QuestionPro survey was disseminated through newsletters, trainings, leadership associations, elected officials, emails listservs, and organizational websites. The University of Tennessee’s institutional review board system (UTK IRB-18-04911-XM) approved this process.

The survey included four sections: consent, demographics, OUD-related strengths and challenges, and OUD-perceived stigma. After reading study details, consent was indicated by continuing on to the survey. Demographics included county of residence, zip code, age, sex, education level, and employment status. Experience of opioid addiction, both personal and for someone close to the respondent, were also assessed. The survey included 19 community strengths and 24 challenges, identified in the literature and supplemented and refined by local working group members. The respondents also had the option to add other responses. Respondents selected the top three challenges and strengths related to a local increase (challenge) or decrease (strength) of OUD or opioid addiction.

Perceived OUD stigma was measured through the eight-item Perceived Stigma of Substance Abuse Scale,^7^ which was adapted to reflect OUD-related stigma. Possible responses spanned from (1) *strongly disagree* to (5) *strongly agree.* A summary score was calculated, with a higher score indicating greater perceived OUD stigma. The original scale had been established with good face and construct validity, and adequate levels of internal consistency.^7^ Statistical analyses were conducted in SPSS v. 24 (Chicago IL). Descriptive analyses were run to gather baseline characteristics and identify the top perceived community strengths and challenges. Independent samples t-tests were run to examine differing stigma levels by people with and without OUD.

## RESULTS

### Participant Characteristics

Of the 577 responses (n_online_=519; n_paper_=58), most respondents were female (n=422, 83.1%), 40–54 years of age (n=178, 34.9%), educated beyond high school (n=427, 83.4%), and employed full-time (n=353, 27.7%). Most respondents had not been addicted to opioids (n=440, 85.3%), but three out of four had someone close to them experience opioid addiction (n=382, 74.0%) (Table 1). The number of respondents by county ranged from nine to 88.

### Strengths and Challenges

The participants’ most frequently selected community challenges were (1) not enough treatment and recovery services (n=247, 42.8%); (2) high cost of OUD treatment (n=177, 30.7%); (3) mental illness (n=146, 25.3%); (4) unemployment (n=116, 20.1%); and (5) lack of support groups to prevent OUD relapse (n=115, 19.9%, Table 2). Three of these five were also prioritized by persons with OUD—not enough treatment and recovery services (n=32, 45.1%); high cost of treatment for OUD (n=24, 33.4%); and mental illness (n=15, 21.1%)—but they additionally selected poor opinion of people with OUD who seek help (n=22, 31.0%); little community knowledge about addiction (n=14, 19.7%); and lack of social support for people with OUD (n=14, 19.7%).

The top five strengths were (1) support for people with OUD to seek help (n=164, 28.4%); (2) law enforcement (n=131, 22.7%); (3) support groups to prevent OUD relapse (n=118, 20.5%); (4) community services work together or collaborate (n=117, 20.3%); and (5) plenty of treatment and recovery services (n=106, 18.4%). People with OUD also prioritized three of these five strengths: support for people with OUD who seek help (n=27, 38.0%); plenty of treatment and recovery services (n=17, 23.9%); and support groups to prevent OUD relapse (n=19; 26.7%), but they additionally prioritized access to drug treatment centers (n=14, 19.7%); good mental health promotion (n=14, 19.7%); and positive treatment outcomes (n=14, 19.7%). Of note, number of treatment and recovery services (n=58, 10.1%) and support groups to prevent OUD relapse (n=24, 4.2%) were prioritized as both a strength and challenge.

### Perceived Stigma

The mean stigma score (eight items, α=0.82) was 29.27 (n=507, range=14–40). People with personal OUD experience (M=30.63, SD=4.67) reported significantly higher mean levels of OUD-related stigma (p<0.05), than those without such experience (M=29.04, SD=5.08; see Table 3). No significant difference in the mean stigma score emerged between people who reported having someone close to them with OUD and those who did not.

## IMPLICATIONS

Community investment in OUD prevention is critical for continued discovery of opportunities related to prioritized health promotion in rural Appalachia. This study examined community strengths and challenges related to OUD in rural counties of East Tennessee. These prioritized issues, particularly when examined alongside stigma and experience with OUD, provide guidance for the development and implementation of culturally relevant, evidence-based prevention approaches.

The communities’ prioritization of lack of treatment and recovery services is consistent with secondary data, which identified only one substance abuse addiction recovery site within the RCORP-ETC region.^11^ Reported consequences include long waiting lines and travel times, which were linked with poorer treatment completion rates.^10^ Thus, increasing the quantity of treatment and recovery services may reduce rural OUD-related challenges. Those with lived OUD experience also emphasized stigmatizing challenges: lack of knowledge, looking down on persons seeking help, and lack of social support. These findings suggest that high levels of perceived OUD-related stigma contribute to the opioid epidemic in rural East Tennessee.

Indeed, people with OUD reported higher mean levels of OUD-related stigma than those without, specifically on items related to self-worth, trust, friendship, and hiring for employment. This variability demonstrates that the views of community members in rural Appalachia are not homogenous and need further exploration. The higher levels of perceived stigma by persons with OUD may reflect high levels of self-stigma, which also can disrupt prevention, treatment, and recovery. Accordingly, stigma-reduction interventions should be carefully adapted to address different levels of OUD stigma and audiences in rural communities. As the survey only assessed perceived stigma, future researchers should investigate differing types of stigma and its intersection with OUD prevention, treatment, and recovery.

Further assessment may be needed to understand the conflicting opinion between community strengths and challenges, as some items were selected as both a strength and challenge—sometimes by the same person. While strong participation was achieved, the sample is not representative of the ten-county region; participant demographics showed higher levels of education and employment than typically found across the region. As such, who represents the community may lead to these differences in opinion and limit the generalizability of results. The administration of both online and paper surveys attempted to mitigate these challenges, as did incorporating community partner feedback during survey development. For example, community members suggested using the language “opioid addiction” over “OUD” in our survey, highlighting the challenge of meeting community culture through the use of stigmatizing language.^8^

Community engagement through planning, implementation, evaluation, and assessment was a critical aspect of the approach and a key principle of community-based research.^12^ Thus, despite a short, one-week survey administration window, the ten-county region supported a high response rate. In sum, these findings highlight the importance of assessment and incorporating community voice to strategically identify and select OUD-related overdose prevention objectives and opportunities.

**SUMMARY BOX**

**What is already known about this topic?** Rural East Tennessee continues to be disproportionately impacted by OUD.

**What is added by this report?** This study identified OUD-related community strengths, challenges, and stigma of Rural East Tennessee Appalachia.

**What are the implications for future research?** Findings indicate people with OUD may experience higher mean levels of OUD-related stigma than those without, specifically on items related to self-worth, trust, friendship, and hiring for employment. These results, along with the community’s concern regarding lack of treatment and recovery services inform the prioritization of areas to be addressed in Appalachia.

## Figures and Tables

**Table 1:** Participant characteristics

Variable	Level	n	%
**Age**	18–25	37	7.3
	26–39	155	30.4
	40–54	178	34.9
	55–64	105	20.6
	65 or over	35	6.9

**Sex**	Male	85	16.7
	Female	422	83.1
	Other	1	0.2

**Educational Level**	Less than high school	8	1.6
	High school graduate	77	15.0
	Some college	113	22.1
	Associate degree	63	12.3
	Technical license	46	9.0
	Bachelor’s degree	95	18.6
	Some graduate school	13	2.5
	Graduate school	97	18.9

**Employment Situation**	Full-time	353	27.7
	Part-time	52	1.6
	Temporary	3	12.2
	Student	23	26.1
	Retired	49	14.9
	Not looking for employment	28	14.9
	Unemployed, looking for employment	33	17.6

**Opioid Addiction (past or present)**	Yes	71	13.8
	No	440	85.3
	Prefer not to answer	5	1.0

**Someone Close to you with Opioid Addiction (past or present)**	Yes	382	74.0
	No	129	25.0
	Prefer not to answer	5	1.0

**Table 2:** Top ten (A) challenges and (B) strengths related to opioid use disorder, n (%)

A.			
Three most important challenges that increase OUD or opioid addiction in your community	Total n=577(%)	No OUD n=440(%)	OUD n=71(%)
Not enough treatment and recovery services	247 (42.8)	197 (44.7)	32 (45.1)
High cost of treatment for OUD	177 (30.7)	131 (29.7)	24 (33.4)
Mental illness	146 (25.3)	113 (25.7)	15 (21.1)
Unemployment	116 (20.1)	91 (20.7)	—
Lack of support groups to prevent OUD relapse	115(19.9)	92 (20.9)	12 (16.9)
Poor treatment outcomes	109 (18.9)	84 (19.1)	—
Lack of knowledge of treatment/ recovery services	101 (17.5)	81 (18.4)	11 (15.5)
Physical or emotional abuse	96 (16.6)	69 (15.7)	13 (18.3)
Little community knowledge about addiction	92 (15.9)	72 (16.4)	14 (19.7)
Location of treatment services	78 (13.5)	59 (13.1)	—
Poor opinion of people with OUD who seek help	—	—	22 (31.0)
Lack of social support for people with OUD	—	—	14 (19.7)
Lack of knowledge of treatment/ recovery services	—	—	11(15.5)

B.			
Three most important strengths that reduce OUD or opioid addiction in your community	Total n=577(%)	No OUD n=440(%)	OUD n=71(%)
Support for people with OUD who seek help	164 (28.4)	124 (28.2)	27 (38.0)
Law enforcement	131 (22.7)	117 (26.6)	—
Support groups to prevent OUD relapse	118 (20.5)	91 (20.68)	19 (26.7)
Community services work together or collaborate	117 (20.3)	103 (23.4)	9 (12.7)
Plenty of treatment/ recovery services	106 (18.4)	78 (17.7)	17 (23.9)
Access to routine healthcare	105 (18.2)	92 (20.9)	—
Insurance pays for inpatient treatment	90 (15.6)	71 (16.1)	13 (18.3)
Positive treatment outcomes	88 (15.3)	69 (15.7)	14 (19.7)
Good mental health promotion	77 (13.3)	—	14 (19.7)
Knowledge of treatment/ recovery services	75 (13.0)	—	13 (18.3)
Access to drug treatment centers	—	73 (16.6)	14 (19.7)
Community knowledge about addiction	—	60 (13.6)	—
Naloxone training	—	—	10 (14.1)

**Table 3:** Perceived stigma by persons with and without OUD

Stigma Scale Questions	OUD Status				
	OUD		Without OUD		
	M	SD	M	SD	p-value
Most people would willingly accept someone who has been treated for OUD as a close friend.*	3.45	1.12	3.21	1.00	<0.05

Most people believe that someone who has been treated for OUD is just as trustworthy as the average citizen.*	4.14	0.92	3.89	0.90	<0.05

Most people would accept someone who has been treated for OUD as a teacher of young children in a public school.*	4.32	0.80	4.17	0.88	0.18

Most people would hire someone who has been treated for OUD to take care of their children.*	4.33	0.68	4.22	0.82	0.29

Most people think less of a person who has been in treatment for OUD.	3.90	1.03	3.48	1.00	<0.05

Most employers will hire someone who has been treated for OUD if he or she is qualified for the job.*	3.44	0.93	3.24	1.02	<0.05

Most employers will pass over the application of someone who has been treated for OUD in favor of another applicant.	3.64	1.11	3.57	1.03	0.60

Most people would be willing to date someone who has been treated for OUD.*	3.39	0.97	3.31	0.93	0.54

* Indicates a reverse-scored item.
